# A prospective cohort register-based study of chronic postsurgical pain and long-term use of pain medication after otorhinolaryngological surgery

**DOI:** 10.1038/s41598-021-84788-4

**Published:** 2021-03-04

**Authors:** Nina Graf, Katharina Geißler, Winfried Meißner, Orlando Guntinas-Lichius

**Affiliations:** 1 Department of Otorhinolaryngology, Jena University Hospital, Am Klinikum 1, 07747 Jena, Germany; 2 Department of Anesthesiology and Intensive Care, Jena University Hospital, Am Klinikum 1, 07747 Jena, Germany

**Keywords:** Quality of life, Risk factors, Oral diseases

## Abstract

Data on chronic postsurgical pain (CPSP) after otorhinolaryngological surgery are sparse. Adult in-patients treated in 2017 were included into the prospective PAIN OUT registry. Patients’ pain on the first postoperative day (D1), after six months (M6) and 12 months (M12) were evaluated. Determining factor for CPSP was an average pain intensity ≥ 3 (numeric rating scale 0–10) at M6. Risk factors associated with CPSP were evaluated by univariate and multivariate analyses. 10% of 191 included patients (60% male, median age: 52 years; maximal pain at D1: 3.5 ± 2.7), had CPSP. Average pain at M6 was 0.1 ± 0.5 for patients without CPSP and 4.2 ± 1.2 with CPSP. Average pain with CPSP still was 3.7 ± 1.1 at M12. Higher ASA status (Odds ratio [OR] = 4.052; 95% confidence interval [CI] = 1.453–11.189; *p* = 0.007), and higher minimal pain at D1 (OR = 1.721; CI = 1.189–2.492; *p* = 0.004) were independent predictors of CPSP at M6. Minimal pain at D1 (OR = 1.443; CI = 1.008–2.064; *p* = 0.045) and maximal pain at M6 (OR = 1.665; CI = 1.340–2.069; *p* < 0.001) were independent predictors for CPSP at M12. CPSP is an important issue after otorhinolaryngological surgery. Better instrument for perioperative assessment should be defined to identify patients at risk for CPSP.

## Introduction

Chronic postsurgical pain (CPSP) is an important clinical problem which significantly influences recovery after surgery and patients’ quality of life^1^. It has been shown for several types of the surgery, that patients with CPSP have a significantly lower physical and mental health, show higher fatigue levels, and less social functioning than patients without CPSP^2–4^. The risk for the development of CPSP still is underestimated in clinical routine and a largely unrecognized clinical problem^5^. Up to and including the International Classification of Diseases (ICD), 10th revision (ICD-10; last update November 2020) did not offer a diagnostic category for the classification of CPSP. The 11th revision of the ICD (ICD-11) will come into effect on 1 January 2022. The International Association for the Study of Pain (IASP) defined CPSP for the ICD-11 as chronic pain that develops or increases in intensity after a surgical procedure or a tissue injury and persists beyond the healing process, i.e., at least 3 months after the surgery^6^. Furthermore, other causes for the pain have been excluded; and the possibility that the pain is from a pre-existing condition has been excluded^7^.

It is said that CPSP occurs in 10–86% of patients after common operations, such as groin hernia repair, breast and thoracic surgery, leg amputation, and coronary artery bypass surgery^5,8^. Severe CPSP is reported for 2–15% of the surgical patients^1,5^. One Norwegian population-based study has been published reporting that 10.5% of surgical patients developed CPSP and still 6.2% after excluding all patients with any pain before surgery^9^.

It is well known that otorhinolaryngological (ORL) and head and neck surgery can lead to severe acute postoperative pain. Even a so-called minor surgery like tonsillectomy is ranked among the 25 procedures with highest pain intensities^10^. ORL and head and neck surgery is underrepresented and neglected as a specific entity in studies on CPSP. Assessment of the multifactorial nature and subjective experience of CPSP has proved challenging for researchers although a number of tools and pain questionnaires are available for the assessment of pain. The assessment should also include the major psychosocial dimensions of pain^11^. Beyond acute postoperative pain, several demographic, psychosocial, genetic factors, as well as preoperative pain, surgical factors, and perioperative anesthesia and analgesia take influence on the risk of development of CPSP^12^.

Using our well-established prospective registry structure of the nationwide project Quality Improvement in Postoperative Pain Treatment (QUIPS; www.quips-project.de) in Germany, the present prospective study used QUIPS data in combination together with questionnaire-based follow-up data to investigate postoperative pain, pain medication use, and pain-related interference up to 12 months after ORL and head and neck surgery.

## Results

### Patients’ characteristics and perioperative process parameters

Baseline characteristics and data at D1 were available for 204 patients. 191 patients answered the questionnaire at M6 and formed the final study group. 13 patients could not be contacted or did not answer (lost to follow-up). Patients’ and surgical characteristics as well as the postoperative pain scores were not different between patients of the final study groups and patients lost to follow-up (Supplementary Table S1 online). The final study group (60% male, median age: 52 years) covered a wide range of typical ORL surgeries (cf. Supplementary Table S1 online). The majority (60%) had no relevant comorbidity. 22% had chronic pain before the ORL surgery. 42% received a perioperative antibiotic treatment. Supplementary Table S2 online shows the anesthetic and analgesic management. The non-opioid and opioid utilization were as follows: Most patients (88.2%) received remifentanil intraoperatively. 52.9% received non-opioids and 15.2% opioids in the recovery room. On ward, 52.9% received non-opioids and 12.3% opioids.

### Postoperative pain at D1, M6, and M12

**Figure 1:**
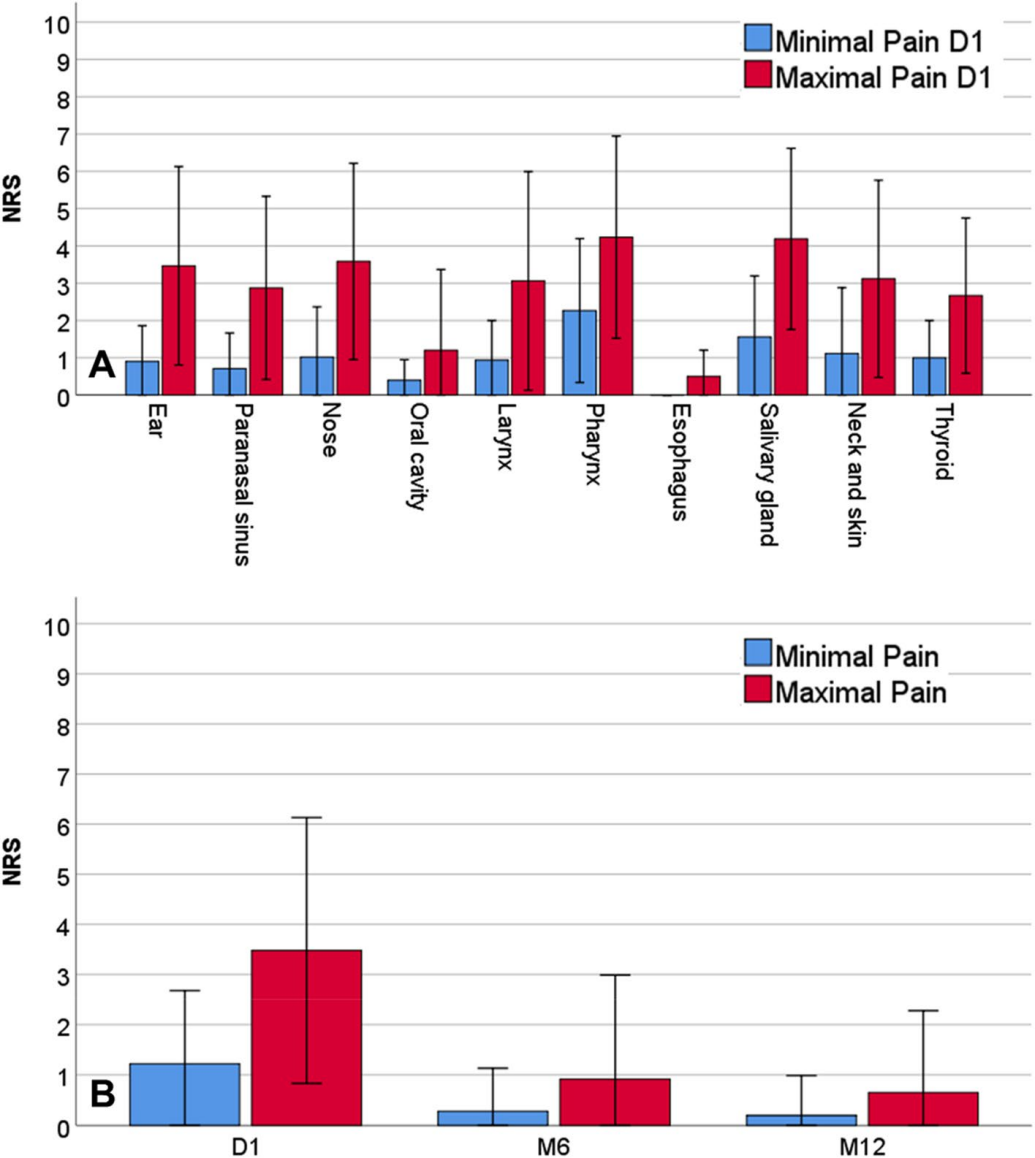
Average minimal and maximal pain at the first operative day (D1), after six months (M6), and after 12 months (M12). **A**: Pain at D1 for the different surgical groups. **B**: Comparison between D1, M6 and M12. NRS = numeric rating scale.

Pain intensity on D1 depended on the type of surgery. Pharynx surgery was the most painful surgery (cf. Fig. 1A). Minimal pain, pain at activity, and maximal pain at D1 under the effect of pain medication were 1.2 ± 1.5, 2.7 ± 2.2, and 3.4 ± 2.6, respectively. Patients with chronic pain already before surgery, reported an average chronic pain intensity of 5.3 ± 2.2 on the numeric rating scale (NRS) before ORL surgery. Overall, minimal and maximal pain decreased significantly at M6 and at M12, respectively (cf. Fig. 1B). Four out of five patients (81.7% and 80.1%, respectively) had no longer any pain at M6 and at M12 (NRS = 0). In the subgroup of patients with persisting pain (independent of the intensity; NRS ≥ 1) under pain medication at M6, minimal, average, and maximal pain were 1.0 ± 1.5, 1.6 ± 0.6, and 4.5 ± 2.5, respectively. At M12, these patients reported under pain medication minimal, average, and maximal pain of 1.0 ± 2.6, 1.6 ± 0.6, and 3.6 ± 2.1, respectively. The results for the subgroup of patients without preoperative chronic pain before surgery and persisting pain (21 patients at M6 and 20 patients at M12) were as follows: Minimal, average, and maximal pain at M6 were 1.1 ± 1.2, 2.5 ± 1.3, and 4.1 ± 2.1, respectively. Minimal, average, and maximal pain at M12 were 0.8 ± 0.9, 1.5 ± 0.5, and 3.2 ± 1.9, respectively.

### CPSP, pain medication and pain-related functional interference at M6 and M12

172 patients out of 191 patients of the final study group (90%) had no CPSP and 19 (10%) had CPSP at M6. The pain at M6 was always localized to the surgical site or referred area, i.e. in the head (11 patients), neck (7 patients), and thorax (1 patient). 5 patients were lost for follow-up at M12. 168 patients had no CPSP (90%) and 18 had CPSP at M12 (10%). At M12, the pain was localized in the head (12 patients), neck (5 patients), and thorax (1 patient). Half of the patients (9 patients) with CPSP at M6 and M12, respectively, had chronic pain before ORL surgery. The localization of the chronic pain before surgery was different from CPSP localization in all cases. At M6 still 22 patients (11.5%) and at M12 still 20 patients (10.5%) took pain analgesics (Supplementary Table S3 online). Three patients (two men, one woman) still took opioids at M6 and at M12. These three patients did not complain of chronic pain and did not take analgesics before the ORL surgery. They underwent surgery for tongue cancer (oral resection and neck dissection), a benign parotid tumor (parotidectomy), and for a cholesteatoma (middle ear surgery).

**Figure 2:**
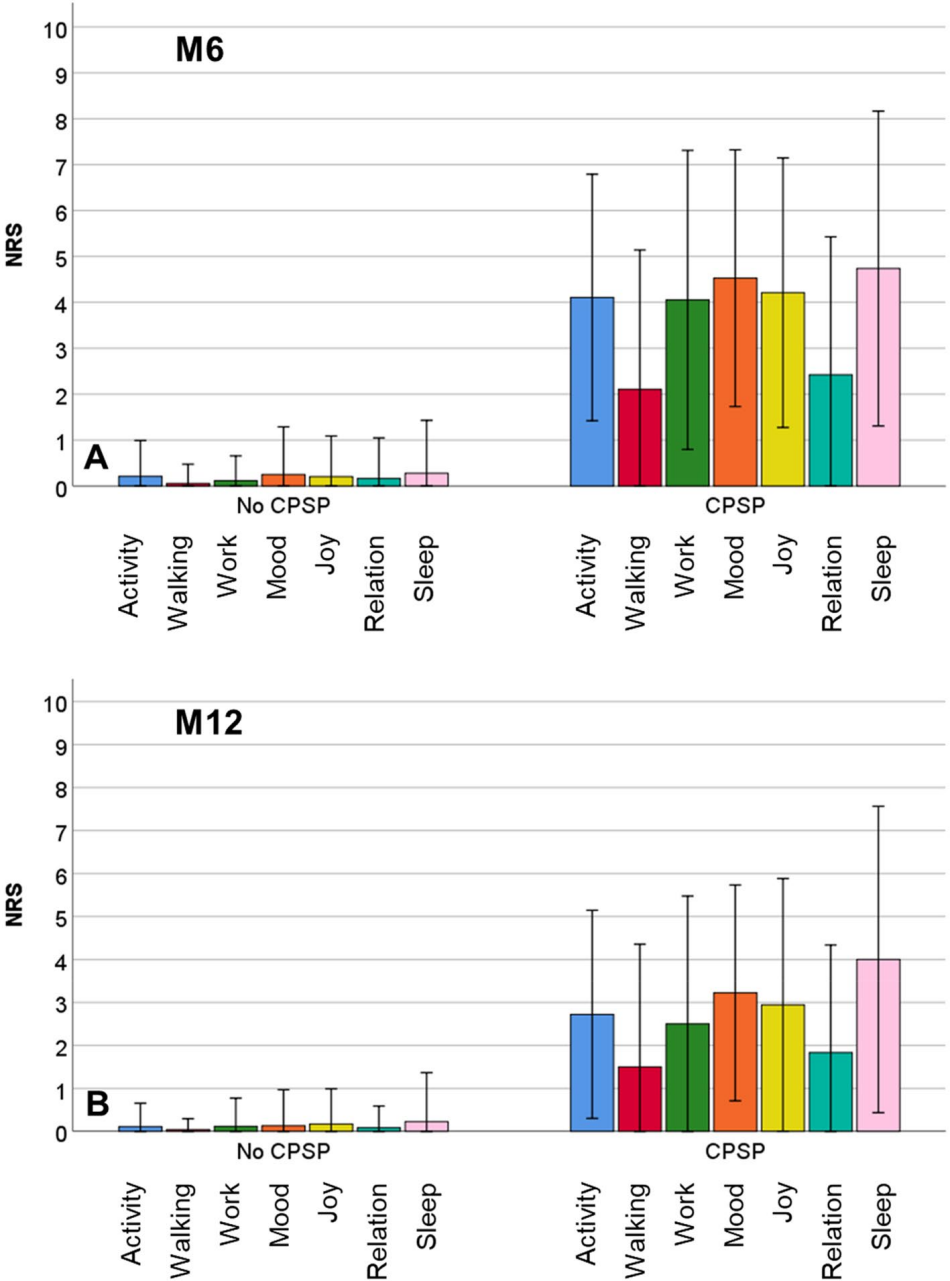
Average functional interference (Physical interference: general activity, walking ability, work; affective interference: mood, enjoyment of life, relations with other persons; and sleep interference) in patients without chronic postsurgical pain (CPSP) and with CPSP at M6 (**A**) and at M12 (**B**). NRS = numeric rating scale.

Figure 2 is showing the functional interference for patients without and with CPSP at M6 and M12. All three domains, physical interference, affective interference, and sleep were affected in patients with CPSP and unaffected in patients without CPSP. Details are presented in Tables 1 and 2. The interference was stronger at M6 compared to M12.

**Table 1:** Comparison of the characteristics of patients without chronic postsurgical pain (CPSP) or with CPSP 6 months (M6) after otorhinolaryngological (ORL) surgery.

All patients	No CPSP	CPSP	p*
	N	N	
All	172	19	
Gender			0.509
Male	104	10	
Female	68	9	
Surgical group			**0.009**
Ear	28	2	
Paranasal sinus	21	3	
Nose	47	1	
Oral cavity	5	0	
Larynx	16	0	
Pharynx	24	6	
Esophagus	2	0	
Salivary glands	13	3	
Neck and skin	15	2	
Thyroid	1	2	
OPS codes			**0.020**
1–40 to 1–58	11	2	
1–61 to 1–69	13	1	
5–01 to 5–05	1	2	
5–06 to 5–07	3	2	
5–18 to 5–20	30	2	
5–21 to 5–22	65	4	
5–23 to 5–28	25	4	
5–29 to 5–31	11	0	
5–40 to 5–41	6	2	
5–42 to 5–54	2	0	
5–16, 5–77	2	0	
5–85, 5–89 to 5–92	3	0	
Side of surgery			0.755
Bilateral	96	11	
Left	39	3	
Right	37	5	
Major surgical complications			NA
Yes	0	0	
No	172	19	
Charlson comorbidity index			0.549
0	98	14	
1	41	4	
2	17	0	
3	9	0	
4	6	1	
5	1	0	
ASA status			**0.006**
I	45	1	
II	101	10	
III	26	8	
Revision surgery			0.805
Yes	7	1	
No	165	18	
Chronic pain before surgery			**0.015**
Yes	38	10	
No	134	9	
Pain medication before surgery			**0.026**
Yes	34	8	
No	138	11	
Opioids before surgery			0.093
Yes	5	2	
No	167	17	
Perioperative antibiotics			0.387
Yes	72	6	
No	100	13	
	Mean ± SD	Mean ± SD	
Age, years	51.2 ± 17.1	57.7 ± 15.5	0.113
Duration of surgery, min	63.2 ± 51.7	79.9 ± 61.3	0.204
Pain at activity (NRS) at D1	2.5 ± 2.1	4.2 ± 2.6	**0.001**
Maximal pain (NRS) at D1	3.3 ± 2.6	4.9 ± 2.6	**0.011**
Minimal pain (NRS) at D1	1.0 ± 1.3	2.6 ± 1.9	**< 0.001**
Satisfaction with pain therapy (NRS) at D1	8.3 ± 2.7	8.8 ± 1.5	0.464
If chronic pain before surgery, intensity (NRS) D1	5.2 ± 2.1	5.7 ± 2.6	0.597
Maximal pain (NRS) at M6	0.1 ± 1.5	5.3 ± 1.8	**< 0.001**
Minimal pain (NRS) at M6	0.1 ± 0.4	2.2 ± 1.5	**< 0.001**
Average pain (NRS) at M6	0.1 ± 0.5	4.2 ± 1.3	**< 0.001**
Actual pain (NRS) at M6	0.1 ± 0.4	3.3 ± 2.0	**< 0.001**
Physical interference (NRS) at M6			
General activity	0.2 ± 0.8	4.1 ± 2.7	**< 0.001**
Walking ability	0.1 ± 0.4	2.1 ± 3.0	**< 0.001**
Work	0.1 ± 0.5	4.1 ± 3.3	**< 0.001**
Affective interference (NRS) at M6			
Mood	0.3 ± 1.0	4.5 ± 2.8	**< 0.001**
Enjoyment of life	0.2 ± 0.9	4.2 ± 2.9	**< 0.001**
Relations with other persons	0.2 ± 0.9	2.4 ± 3.0	**< 0.001**
Sleep interference (NRS) at M6	0.3 ± 1.2	4.7 ± 3.4	**< 0.001**

NRS = numeric rating scale; OPS = Operation and Procedure Code. *Pearson’s chi-square test or Fisher’s exact test was used to compare ordinal and nominal data of patients without CPSP and with CPSP. The nonparametric Mann–Whitney-U-test was used to compare metric data.

Bold values are statistically significant for *p* < 0.05.

**Table 2:** Comparison of the characteristics of patients without chronic postsurgical pain (CPSP) or with CPSP 12 months (M12) after otorhinolaryngological (ORL) surgery.

All patients	No CPSP	CPSP	p*
	N	N	
All	168	18	
Gender			0.935
Male	101	11	
Female	67	7	
Surgical group			0.113
Ear	28	1	
Paranasal sinus	20	4	
Nose	45	2	
Oral cavity	5	0	
Larynx	15	1	
Pharynx	26	3	
Esophagus	2	0	
Salivary glands	11	5	
Neck and skin	13	2	
Thyroid	3	0	
OPS codes			0.336
1–40 to 1–58	10	1	
1–61 to 1–69	12	2	
5–01 to 5–05	2	1	
5–06 to 5–07	5	0	
5–18 to 5–20	30	1	
5–21 to 5–22	63	5	
5–23 to 5–28	25	4	
5–29 to 5–31	10	1	
5–40 to 5–41	5	2	
5–42 to 5–54	2	0	
5–16, 5–77	1	1	
5–85, 5–89 to 5–92	3	0	
Side of surgery			0.588
Bilateral	96	8	
Left	36	5	
Right	36	5	
Major surgical complications			NA
Yes	0	0	
No	168	18	
Charlson comorbidity index			0.915
0	95	12	
1	41	4	
2	16	1	
3	8	1	
4	7	0	
5	1	0	
ASA status			0.116
I	45	1	
II	94	14	
III	29	3	
Revision surgery			0.783
Yes	7	1	
No	161	17	
Chronic pain before surgery			**0.007**
Yes	36	9	
No	132	9	
Pain medication before surgery			0.537
Yes	36	5	
No	132	13	
Opioids before surgery			0.085
Yes	5	2	
No	163	16	
Perioperative antibiotics			0.820
Yes	98	11	
No	70	7	
	Mean ± SD	Mean ± SD	
Age, years	50.8 ± 17.3	57.2 ± 13.9	0.129
Duration of surgery, min	62.5 ± 51.2	85.2 ± 64.9	0.094
Pain at activity (NRS) at D1	2.5 ± 2.2	4.3 ± 2.2	**0.001**
Maximal pain (NRS) at D1	3.3 ± 2.6	4.7 ± 2.4	**0.027**
Minimal pain (NRS) at D1	1.0 ± 1.4	2.4 ± 1.8	**< 0.001**
Satisfaction with pain therapy (NRS) at D1	8.4 ± 2.6	7.8 ± 2.4	0.368
If chronic pain before surgery, intensity (NRS) D1	5.3 ± 2.2	5.9 ± 2.3	0.437
Maximal pain (NRS) at M6	0.1 ± 1.5	5.2 ± 1.7	**< 0.001**
Minimal pain (NRS) at M6	0.1 ± 0.4	2.1 ± 1.5	**< 0.001**
Average pain (NRS) at M6	0.1 ± 0.5	4.2 ± 1.2	**< 0.001**
Actual pain (NRS) at M6	0.1 ± 0.4	3.3 ± 2.0	**< 0.001**
Maximal pain (NRS) at M12	0.2 ± 0.7	4.9 ± 1.7	**< 0.001**
Minimal pain (NRS) at M12	0.1 ± 0.2	1.8 ± 1.9	**< 0.001**
Average pain (NRS) at M12	0.1 ± 0.3	3.7 ± 1.1	**< 0.001**
Actual pain (NRS) at M12	0.1 ± 0.2	2.1 ± 0.5	**< 0.001**
Physical interference (NRS) at M12			**< 0.001**
General activity	0.1 ± 0.6	2.7 ± 2.4	**< 0.001**
Walking ability	0.0 ± 0.3	1.5 ± 2.9	**< 0.001**
Work	0.1 ± 0.7	2.5 ± 3.0	**< 0.001**
Affective interference (NRS) at M12			
Mood	0.1 ± 0.8	3.2 ± 2.5	**< 0.001**
Enjoyment of life	0.2 ± 0.8	2.9 ± 2.9	**< 0.001**
Relations with other persons	0.1 ± 0.5	1.8 ± 2.5	**< 0.001**
Sleep interference (NRS) at M12	0.2 ± 1.1	4.0 ± 3.6	**< 0.001**

NRS = numeric rating scale. *Pearson’s chi-square test or Fisher’s exact test was used to compare ordinal and nominal data of patients without CPSP and with CPSP. The nonparametric Mann–Whitney-U-test was used to compare metric data.

Bold values are statistically significant for *p* < 0.05.

### Comparison of the characteristics of patients without CPSP and with CPSP at M6 and at M12

Table 1 is summarizing the comparison of patients without CPSP and with CPSP at M6. CPSP was related to pharyngeal and thyroid surgery (*p* = 0.009), higher ASA status (*p* = 0.006), chronic pain before surgery (*p* = 0.015), and pain medication intake before surgery (*p* = 0.026). All functional interference parameters were worse in patients with CPSP (all *p* < 0.0001). Age, gender and other patient factors had no influence (all *p* > 0.05). From all pain parameters at D1 (minimal, maximal, pain in activity), minimal pain showed the largest difference between patients without versus with CPSP. Table 2 is showing the data at M12. Chronic pain before surgery was the only remaining factor with association to CPSP (*p* = 0.007). Still all functional interference parameters were worse in patients with CPSP (all *p* < 0.0001). From all pain parameters measured at D1, still minimal pain was the best distractor between patients without versus with CPSP. Beyond average pain (defining CPSP), also minimal and maximal pain at M6, were significantly different between patients without versus with CPSP at M12.

In the multivariate analysis, the ASA status (Odds ratio [OR] = 4.052; 95% confidence interval [CI] = 1.453–11.189; *p* = 0.007), only thyroid surgery (OR = 31.202; CI = 1.498–650.055; *p* = 0.026), and minimal pain at D1 (OR = 1.721; CI = 1.189–2.492; *p* = 0.004) remained independent risk factor for CPSP at M6 (Table 3). Chronic pain before surgery was not an independent risk factor for CPSP at M6. Two multivariate analyses were performed for M12, once including minimal pain at M6 (model 1), then maximal pain at M6, into the analysis (model 2; Table 4). In model 1, minimal pain at M6 remained as the only independent predictor for CPSP at M12 (OR = 2.889; CI = 1.669–5.000; *p* < 0.001). In model 2, minimal pain at D1 (OR = 1.443; CI = 1.008–2.064; *p* = 0.045) and maximal pain at M6 (OR = 1.665; CI = 1.340–2.069; *p* < 0.001) remained independent predictors for CPSP at M12. Also at M12, chronic pain before surgery was not an independent risk factor for CPSP.

**Table 3:** Multivariate binary logistic regression analysis of risk factors for CPSP at 6 months (M6).

Parameter	OR	Lower 95% CI	Upper 95% CI	*p*
Surgical group				
Ear	1			
Paranasal sinus	2.502	0.324	9.335	0.379
Nose	0.292	0.022	3.851	0.350
Oral cavity	0.000	0.000		0.999
Larynx	0.000	0.000		0.998
Pharynx	1.061	0.145	7.744	0.954
Esophagus	0.000	0.000		0.999
Salivary glands	1.672	0.173	16.147	0.657
Neck and skin	1.054	0.108	10.252	0.964
Thyroid	31.202	1.498	650.055	**0.026**
ASA status	4.032	1.453	11.189	**0.007**
Chronic pain before surgery				0.340
No	1			
Yes	1.859	0.520	6.650	
Minimal pain (NRS) at D1	1.721	1.189	2.492	**0.004**

NRS = numeric rating scale.

Bold values are statistically significant for *p* < 0.05.

**Table 4:** Multivariate binary logistic regression analysis of risk factors for CPSP at 12 months (M12).

Parameter	OR	Lower 95% CI	Upper 95% CI	*p*
**Model 1**				
Chronic pain before surgery	1.484	0.411	5.352	0.547
Yes				
No				
Minimal pain (NRS) at D1	1.361	0.959	1.933	0.085
Minimal pain (NRS) at M6	2.889	1.669	5.000	**< 0.001**
**Model 2**				
Chronic pain before surgery	1.489	0.406	5.465	0.548
Yes				
No				
Minimal pain (NRS) at D1	1.443	1.008	2.064	**0.045**
Maximal pain (NRS) at M6	1.665	1.340	2.069	**< 0.001**

NRS = numeric rating scale.

Bold values are statistically significant for *p* < 0.05.

## Discussion

A large and representative ORL cohort of 191 patients was analyzed for CPSP up to 12 months after typical ORL and head and neck surgery and the typical spectrum of postoperative pain at D1. Based on a prospective register and an excellent 94% response rate, 18% of the patients had pain at M6 and 20% at M12. Most important, 10% fulfilled the criteria of CPSP at M6 and at M12. If we put aside all patients with chronic pain before ORL surgery, still 5% of patients remain with CPSP. CPSP was associated with important pain-related interferences. Higher ASA status and higher pain at D1 were independent predictors of CPSP at M6. Pain at D1 and persisting pain at M6 were independent predictors for CPSP at M12. Chronic pain before surgery was not an independent predictor of CPSP. The localization of the chronic pain before surgery was different from CPSP localization in all cases implying that the occurrence of chronic pain after surgery is not the continuation of the preexisting chronic pain. The preexistent chronic pain in another body region was not suggestive for the development of CPSP.

ORL and head and neck surgery were so far underrepresented in CPSP studies. A data analysis of 3120 patients from the European registry included 60 patients after thyroidectomy^1^. Equivalent to the present study, 11% of all cases and 13% of the thyroidectomy cases in this European study developed CPSP, but thyroidectomy was no risk factor for CSPS at M12. A recent study of the PAIN OUT registry with 2322 patients reported the development of CPSP in 15.3% at M12, but did not include ORL cases^13^. In a cross-sectional survey from 2007 to 2008 in Norway with 2043 participants with surgery 3 months to 3 years preceding the survey, 18.3% reported persistent moderate or severe pain^9^. Out of this, patients with head, face, neck, throat surgery (not specified more exactly) reported in 17–22% of persistent mild to severe pain (not differentiated). In the present study, patients with pharyngeal surgery were the largest group of patients with CPSP at M6. It is well known that pharyngeal surgery can be followed by sever postoperative pain^10,14^. Hence, it fits together that pain at D1 was associated to CPSP at M6 and M12. In conclusion, the presented series seems to be the largest study exclusively for ORL and head and neck surgery, and a CPSP rate of about 10% seems to be plausible.

At M6 and M12 still 11.5% and 10.5%, respectively, of the patients (all were CPSP patients), took pain medication. There is increasing data that perioperative opioid use is an important risk factor for long-term opioid dependence. In the present study, only 11.8% of the patients received perioperative opioid use and the application was not a risk factor for CPSP. It is reported that 5–10% of surgical patients exhibit a persistent postoperative opioid use^15^. Hence, opioid dependence got much attention in the media. In contrast, not much is known about long-term postoperative non-opioid dependence. In the present study, three patients (1.6%) still took opioids. None of these three patients was a head and neck cancer patient. Nearly to one-third of head and neck cancer patients still is taking opioids 6–12 months after surgery^16,17^. In a recent population-based cohort study among patients undergoing plastic and reconstructive surgery, a persistent opioid use occurred in 6.6% of the patients^15^. A recent analysis of a U.S. nationwide insurance claims dataset from 2013 to 2014 analyzed persistent opioid use after minor and major surgery^18^. From head and neck surgery, thyroidectomy patients were included. Thyroidectomy patients had an incidence of 5.1% of new persistent opioid use within 90–180 days after surgery. In comparison to the U.S. American data, the incidence of long-term opioid dependence was low in the present study. This may be due to a more restrictive prescription of opioids in Germany. The present study did not include children. Tonsillectomy is one of the most painful ORL surgeries and is mainly performed in children and young adults. A recent analysis of a database of a large national private insurer in the U.S. showed that many children receive postoperative opioid prescription fills^19^. Therefore, it would be worthwhile to investigate the incidence of CPSP after pediatric ORL and head and neck surgery.

Higher comorbidity as reflected by the ASA status, higher pain at D1, persisting pain at M6 were independent predictors for CPSP at M12. Preoperative chronic pain was not an independent predictor in the present series. The multivariate analysis of the PAIN OUT trial identified orthopedic surgery, preoperative chronic pain and percentage of time in severe pain on D1 as risk factors for M12^1^. Younger age, body mass index, psychological distress were predictors for persistent post-surgical pain (NRS ≥ 1) in the already mentioned cross-sectional survey^9^. This makes clear that the immediate postoperative pain and probably many psychosocial factors that were not analyzed so far, have a major impact on the risk to develop CPSP. It is well known that psychological factors like preoperative anxiety, the pain catastrophizing level, and resilience have a major impact on postoperative pain after ORL surgery^20^. It is likely that these factors have direct or indirect impact also on the risk to develop CPSP. Furthermore, pain intensity can be considered as one determining key factor for CPSP. Many of the mentions psychosocial factors contribute to the bio-psycho-social model of chronic pain. This should be analyzed in future studies. The characteristics and quality of the chronic postoperative pain should be analyzed in more detail as well as the impact of chronic pain on physical and mental health.

In the present study, the patients in the CPSP group were older. The difference was not statistically significant but a trend was seen. The ratio of the elderly patients scheduled for ORL surgery is presumably growing in the future. Frailty, delirium, and cognitive deficit are regarded as further important factors in the development of chronic post-surgical pain in older adults^21^. Older patients seem to need specific chronic post-surgical pain treatment and prevention strategies.

As usual the present study was based on patient self-report, the outcomes assessed by validated questionnaires. Although this work was not a randomized controlled study and the authors did not focus on the exact analgesic dose regimens, moreover, the pain management was not standardized because of the vast diversity of patients, illnesses, and surgical interventions which can be a limitation of this research, the results are consistent with former investigations regarding the effective pain management, lower opioid use, greater patients’ satisfaction, and low CPSP rate.

Only 5% of patients remained with CPSP due to the multimodal perioperative pain management, which seems to be one of the most important factors in the prevention of CPSP. In the present study, only 11.8% of the patients received perioperative opioid medication. Opioid-free anesthesia has gained in popularity as a way to enhance early recovery and to spare opioids for the postoperative period^22,23^. Future trials should therefore focus on the principles of opioid-free anesthesia in ORL surgery for preventing adverse side effects of opioids and dependence. The presented study based on the assessment of the pain intensity and analgesic medication can be the basis for further investigations for a better understanding of the development of CPSP after ORL surgery. These studies should standardize or randomize the perioperative and postoperative opioid prescription to analyze the effects on chronic postsurgical pain. The effective and satisfactory pain management seems to be the most important factor in the prevention of CPSP since the intensity and duration of the pain can be considered as the main determining key factors for the development of CPSP.

Data on the course of the individual (acute versus chronic) diseases were not analyzed. Especially, pain characteristics in patients with head and neck cancer or other severe chronic ORL diseases may have affected the results at M6 and M12. Moreover, preoperative psychosocial factors like tobacco, alcohol and substance abuse disorders, mood disorders, and anxiety are known risk factors especially for persistent opioid use after surgery^18^. Assessment of such preoperative factors should be included in future trials.

In conclusion, this prospective cohort register-based study on 191 patients representing everyday clinical practice after otolaryngological and head and neck surgery showed that a considerable number of patients develop CPSP and therefore take analgesics. Patients at high risk for CPSP need to be identified early and offered coordinated and comprehensive prevention and perioperative care at best by a multidisciplinary team consisting of pain physicians, advanced practice nurses, psychologists, and physiotherapists^24^. Such special perioperative and postoperative pain services are so far offered only at a few places^25^.

## Methods

### Ethical considerations

The present prospective cohort study was part of the PAIN OUT (Improvement in postoperative PAIN OUTcome; www.pain-out.eu) project and theGerman-wide Quality Improvement in Postoperative Pain Treatment (QUIPS; www.quips-project.de) registry (German Clinical Trials Register DRKS00006153). The ethics committee of the Jena University Hospital, Jena, Germany, approved the study protocol for a data collection of routine and anonymized hospital data as well as the QUIPS questionnaires. The study followed all relevant guidelines of the institutional ethics committee and the principles of the Declaration of Helsinki. By completing the patient questionnaire, a patient gave written informed consent for study participation.

### QUIPS registry and inclusion criteria

Data collection within the QUIPS registry reflects clinical practice in perioperative pain management. Patients scheduled as inpatients for ORL surgery between mid-January and mid-July 2017 (6 months) in the ENT department of Jena University Hospital were included. Other inclusion criteria were age ≥ 18 years, oriented and awake, linguistic and intellectual understanding of German language, written consent.

The QUIPS questionnaires are presented in detail elsewhere^26^. Briefly, the QUIPS questionnaires consisted of two parts: This first part was covering the patient-reported outcome (PRO) parameters and comprised a validated 15-item questionnaire, whereas the second part was filled by the investigator. After a standardized instruction, the patient herself or himself completed the first part at the first postoperative day (D1). QUIPS used 11-point NRS to estimate the patient’s pain intensity during activity, minimal and maximal pain. Generally, higher numbers were indicating more pain (0 = no pain; 10 = most imaginable pain). Furthermore, the patient was asked by dichotomized (yes/no) questions about pain-related impairments (mobility, breathing, sleep, mood), side effects of pain treatment (drowsiness, nausea, vomiting), and satisfaction with the pain management. The second part, which was filled by the investigators, was covering the relevant demographic and clinical parameters like age, gender, type of surgery including coding of the surgical procedures (OPS codes) due to the German procedure classification (OPS-301), anesthesia, pain management, and was collected from the patients’ charts. The patients’ pre-anesthesia overall health status was preoperatively assessed by The American Society of Anesthesiologists physical status classification system (ASA status)^27^. The Charlson comorbidity index (CCI) was utilized to assess the patients’ comorbidity^28^.

### Patient assessment at 6 and 12 months after surgery

Depending on their preferences, patients were contacted via telephone or email after 6 months (M6) and once more after 12 months (M12) with the identical QUIPS questionnaire. If contacted by telephone, the questions of the QUIPS questionnaire were answered by telephone interview. If contacted via email, the patients received an individual code to log into the QUIPS webpage (http://www.quips-projekt.de). Here, the patients answered the same questionnaire. If a patient was not reached via telephone or email, the questionnaire was sent to the patient by regular post. To assess the postsurgical pain, i.e. explicitly related to the ORL surgery, again 11-point NRS were used to estimate the patient’s minimal, maximal pain, and average pain within the last 24 h as well the actual pain. Again, higher numbers were indicating more pain (0 = no pain; 10 = most imaginable pain). All pain drugs taken were listed. Finally, German version of the Brief Pain Inventory (BPI) was used to estimate the functional interference within the last 24 h with three domains: physical interference (general activity, walking ability, and work), affective interference (mood, enjoyment of life, and relations with other persons), and sleep was assessed also using 11-point NRS (0 = no impairment; 10 = maximal impairment)^13,29^. Patient with any intensity of persistent pain (NRS ≥ 1) were differentiated from patient with CPSP: All patients who indicated average pain with NRS ≥ 3 at M6 and/or M12 were defined as patients with CPSP. Patients with average pain NRS < 3 at M6 and/ or M12 were defined as patients without CPSP. Furthermore, the group of patients with CPSP was subdivided into patients without chronic pain of other reasons before surgery and patient with chronic pain before surgery.

### Statistical analysis

If not indicated otherwise, data is presented with mean values ± standard deviation (SD). All statistical analyses were performed using IBM SPSS, version 25. Pearson’s chi-square test or Fisher’s exact test was used to compare ordinal and nominal data of patients without CPSP and with CPSP. The nonparametric Mann–Whitney-U-test was used to compare metric data. Parameters with significant differences between both groups in univariate testing (*p* < 0.05) were included into multivariate binary logistic regression analyses for analysis of independent predictors for CPSP at M6 and at M12. From the pain parameters, minimal pain was included into the multivariate analyses, because minimal pain showed the largest difference between patients without CPSP and with CPSP. Nominal p values of two-tailed tests are reported. The significance level for the multivariable analyses was also set at *p* < 0.05.

## Supplementary Information

Supplementary Information

## Data Availability

The datasets used and/or analyzed during the current study are available from the corresponding author upon reasonable request.
