# Supplementary material for: A prospective cohort register-based study of chronic postsurgical pain and long-term use of pain medication after otorhinolaryngological surgery
Source: Sci Rep. 2021 Mar 4;11:5215. doi: 10.1038/s41598-021-84788-4 (PMC7933142; doi:10.1038/s41598-021-84788-4)

**Supplementary Tables S1-S3 and Supplementary Figure S1**

**A prospective cohort register-based study of chronic postsurgical pain and long-term use of pain medication after otolaryngological surgery**

Nina Graf^1^, Katharina Geißler^1^, Winfried Meißner^2^, Orlando Guntinas-Lichius^1^

^1^ Department of Otorhinolaryngology, Jena University Hospital, Jena, Germany

^2^ Department of Anesthesiology and Intensive Care, Jena University Hospital, Germany

**Supplementary Table S1**

| **Supplementary Table S1.** Patients’ and surgical characteristics in patients of the final study group compared to patients lost to follow-up at 6 months (M6) after otorhinolaryngological (ORL) surgery | | | | | |
| --- | --- | --- | --- | --- | --- |
| All patients | All patients | Final study group | Lost to follow up | p |  |
|  | N | N | N |  |  |
| All | 204 | 191 | 13 |  |  |
| Gender  Male  Female | 123  81 | 114  77 | 9  4 | 0.496 |  |
| Surgical group  Nose  Pharynx  Ear  Paranasal sinus  Neck and skin  Larynx  Salivary glands  Oral cavity  Esophagus  Thyroid | 49  33  32  25  19  18  17  5  3  3 | 48  30  30  24  17  16  16  5  2  3 | 1  3  2  1  3  2  1  0  1  0 | 0.573 |  |
| OPS codes  5-21 to 5-22  5-18 to 5-20  5-23 to 5-28  1-61 to 1-69  1-40 to 1-58  5-29 to 5-31  5-40 to 5-41  5-06 to 5-07  (5-01 to 5-05  5-42 to 5-54  5-85, 5-89 to 5-92  5-16, 5-77 | 71  34  31  16  14  12  10  5  3  3  3  2 | 69  32  29  14  13  11  8  5  3  2  3  2 | 2  2  2  2  1  1  2  0  0  1  0  0 | 0.506 |  |
| Side of surgery  Bilateral  Left  Right | 114  44  46 | 107  42  42 | 7  2  4 | 0.714 |  |
| Major surgical complications  Yes  No | 0  204 | 0  191 | 0  13 | NA |  |
| Charlson comorbidity index  0  1  2  3  4  5 | 116  51  19  10  7  1 | 112  45  17  9  7  1 | 4  6  2  1  0  0 | 0.362 |  |
| ASA status  I  II  III | 48  120  36 | 46  111  34 | 2  9  2 | 0.709 |  |
| Revision surgery  Yes  No | 8  196 | 8  183 | 0  13 | 0.452 |  |
| Prior chronic pain independent from current surgery  Yes  No | 49  155 | 47  144 | 2  11 | 0.451 |  |
| Permanent pain medication taken before and independent from current surgery  Yes  No | 45  159 | 42  149 | 3  10 | 0.927 |  |
| Opioids taken before and independent from current surgery  Yes  No | 8  196 | 7  184 | 1  12 | 0.469 |  |
| Perioperative antibiotics  Yes  No | 82  122 | 78  113 | 4  9 | 0.474 |  |
|  | Mean±SD | Mean±SD | Mean±SD |  |  |
| Age, years | 51.8±17.0 | 51.8±17.0 | 51.0±17.0 | 0.871 |  |
| Duration of surgery, min | 63.4±52.1 | 64.94±52.8 | 39.0±31.4 | 0.110 |  |
| Pain at activity (NRS) at D1 | 2.7±2.2 | 2.7±2.2 | 3.5±2.5 | 0.167 |  |
| Maximal pain (NRS) at D1 | 3.5±2.7 | 3.4±2.6 | 4.2±2.8 | 0.345 |  |
| Minimal pain (NRS) at D1 | 1.2±1.4 | 1.2±1.5 | 1.9±1.3 | 0.101 |  |
| Satisfaction with pain therapy (NRS) | 8.3±2.6 | 8.4±2.6 | 7.8±3.0 | 0.430 |  |
| If chronic pain before surgery, intensity (NRS) | 5.3±2.2 | 5.3±2.2 | 4.5±3.5 | 0.608 |  |

NA=not applicable; D1 = first postoperative day

**Supplementary Table S2**

| **Supplementary Table S2.** Process parameters | | | |
| --- | --- | --- | --- |
| Parameter | N | % | Dosage per day |
| Premedication  No  Midazolam  Clonidine | 21  179  4 | 10.3  87.7  2.0 | 6.34±1.74 mg  150 mg |
| General anesthesia  Yes  No | 199  5 | 97.5  2.5 |  |
| Local anesthesia  Yes  No | 3  201 | 1.5  98.5 |  |
| PONV prophylaxis  Yes  No | 179  25 | 87.7  12.3 |  |
| Remifentanil intraoperatively  Yes  No | 180  24 | 88.2  11.8 |  |
| Clonidine intraoperatively  Yes  No | 1  203 | 0.5  99.5 |  |
| Ketamine intraoperatively  Yes  No | 1  203 | 0.5  99.5 |  |
| Non-opioid in recovery room  No  Metamizole  Acetaminophen | 193  8  3 | 94.6  3.9  1.5 | 1000 mg I.V.  1000 mg I.V. |
| Opioid in recovery room  No  Piritramide | 173  31 | 84.8  15.2 | 5.7±2.28 mg I.V. |
| Non-opioid on ward  No  Ibuprofen  Metamizole  Gabapentin  Etoricoxib | 96  53  52  2  1 | 47.1  26.0  25.5  1.0  0.5 | 919.23±410.18 mg P.O.  2250.00±866.03 mg P.O.  900 mg P.O.  60 mg P.O. |
| Non-opioid on ward  Yes  No | 108  96 | 52.9  47.1 |  |
| Opioid on ward  No  Tramadol  Piritramide  Tilidine  Oxycodone  Morphine | 180  13  4  3  3  1 | 88.2  6.4  2.0  1.5  1.5  0.5 | 78.46±18.19 mg P.O.  3.75±1.6 mg I.V.  83.33±28.87 mg P.O.  20 mg P.O. or I.V.  60 mg P.O. |
| Opioid on ward  Yes  No | 24  180 | 11.8  88.2 |  |
| Ice pack on ward  Yes  No | 38  166 | 18.6  81.4 |  |

P.O. = per os; I.V. = intravenous

**Supplementary Table S3**

| **Supplementary Table S3.** Overview of long-term use of pain medication after 6 months (M6) and 12 months (M12). | | | |
| --- | --- | --- | --- |
|  | M6 | M12 | Usual daily dosage range of the medication |
| No | 169 | 171 |  |
| Acetylsalicylic acid | 1 | 1 | 1500 mg |
| Ibuprofen | 7 | 3 | 1200 – 1600 mg |
| Metamizole | 11 | 8 | 500 – 1000 mg |
| Tramadol retard, WHO step 2 | 2 | 2 | 200 mg |
| Morphine, WHO step 3 | 1 | 1 | 60 mg |
| No answer | 0 | 5 |  |

**Supplementary Figure**

**Supplementary Figure 1.** Flowchart showing the exclusion and final inclusion of the patients with absolute numbers. CPSP, chronic postsurgical pain; M6, after 6 months; M12, after 12 months.


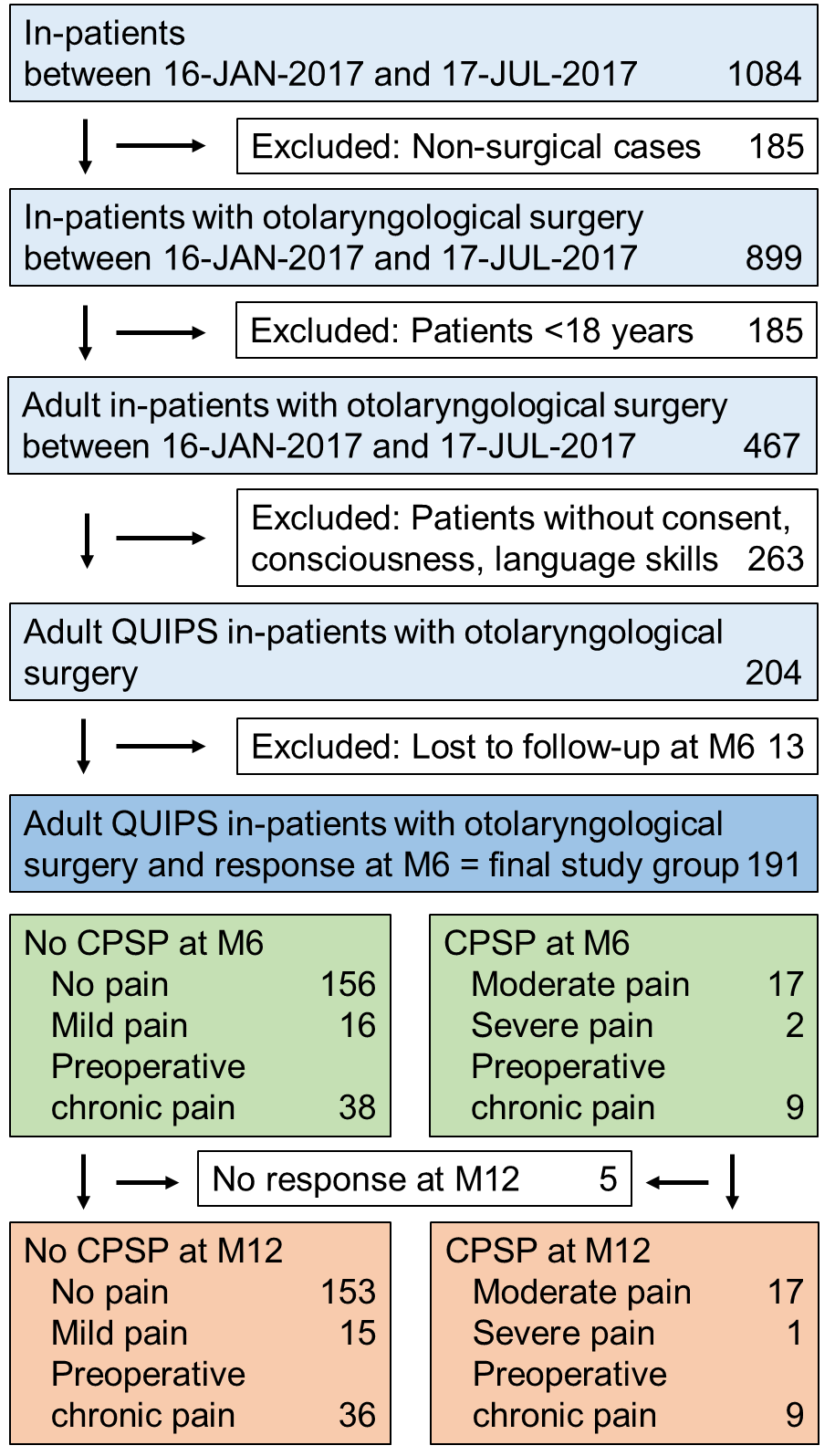

Supplement: Supplementary file 1 — Supplementary Information [file 41598_2021_84788_MOESM1_ESM.docx]
